# Morphological plasticity of the coral skeleton under CO_2_-driven seawater acidification

**DOI:** 10.1038/ncomms8368

**Published:** 2015-06-12

**Authors:** E. Tambutté, A. A. Venn, M. Holcomb, N. Segonds, N. Techer, D. Zoccola, D. Allemand, S. Tambutté

**Affiliations:** 1 Marine Biology Department, Centre Scientifique de Monaco, 8 Quai Antoine 1^er^, Monaco 98000, Monaco; 2 Laboratoire Européen Associé 647 « BIOSENSIB », Centre Scientifique de Monaco- Centre National de la Recherche Scientifique, 8 Quai Antoine 1^er^, Monaco 98000, Monaco

## Abstract

Ocean acidification causes corals to calcify at reduced rates, but current understanding of the underlying processes is limited. Here, we conduct a mechanistic study into how seawater acidification alters skeletal growth of the coral *Stylophora pistillata*. Reductions in colony calcification rates are manifested as increases in skeletal porosity at lower pH, while linear extension of skeletons remains unchanged. Inspection of the microstructure of skeletons and measurements of pH at the site of calcification indicate that dissolution is not responsible for changes in skeletal porosity. Instead, changes occur by enlargement of corallite-calyxes and thinning of associated skeletal elements, constituting a modification in skeleton architecture. We also detect increases in the organic matrix protein content of skeletons formed under lower pH. Overall, our study reveals that seawater acidification not only causes decreases in calcification, but can also cause morphological change of the coral skeleton to a more porous and potentially fragile phenotype.

## Abstract (web summary)

Ocean acidification is predicted to cause major reductions in coral calcification rates. Here, Tambutté *et al*. show that in the coral, reduced calcification rates under low pH are the result of changes in skeletal morphology, rather than skeletal dissolution.

## Graphical abstract

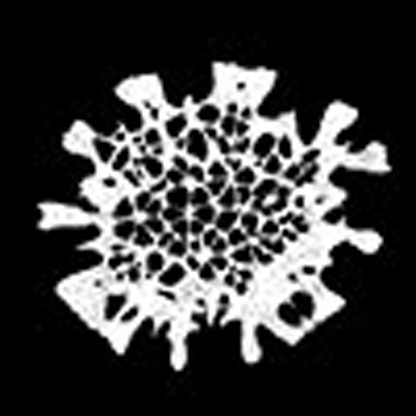

Calcification by scleractinian corals is a major contributor to the structural foundation of coral-reef ecosystems, which harbour an estimated one-quarter to a third of biodiversity in the ocean^1^. Climate change-related phenomena that impinge on the capacity of corals to calcify are therefore considered to be major threats to these marine resources. Declines in seawater pH and associated decreases in CaCO_3_ mineral saturation states (Ω) driven by seawater uptake of CO_2_ (ocean acidification) are predicted to be unfavourable to marine calcification^2^,^3^. An ever-growing body of laboratory and field-based research supports this prediction, and meta-analysis of data derived from such studies indicates declines in coral calcification of 15 to 22 % at levels of ocean acidification predicted to occur under business-as-usual scenarios of CO_2_ emissions by the end of this century^4^.

Nevertheless, there remains uncertainty about the extent of the threat of ocean acidification to coral calcification^5^. Some of this uncertainty stems from the lack of understanding of how and why ocean acidification affects coral skeleton formation^6^. This is partly due to the limited number of mechanistic studies that have attempted to dissect the effects of ocean acidification on multiple aspects of coral growth^7^. Calcification rate (mass addition) is a product of skeletal density (mass unit per volume) and extension (linear growth)^8^, but ocean acidification studies rarely consider both of the latter two parameters. Furthermore, these parameters may vary due to morphological plasticity of the coral skeleton under different environmental regimes^9^. An integrated understanding of how ocean acidification affects coral skeleton formation is crucially needed to form a clearer perspective on physiological tradeoffs or processes of acclimatization that corals could undertake in an ocean with reduced pH and Ω_*aragonite*_.

Here we aim to provide a holistic picture of the impact of ocean acidification on coral skeleton formation, against a backdrop of coral physiology, to provide mechanistic insight into the vulnerability of the coral calcification process to reduced pH and Ω_*aragonite*_. We conducted a controlled laboratory study on *Stylophora pistillata*, which has been used extensively for investigations into mechanisms of coral resilience to ocean acidification^10^,^11^,^12^,^13^ and for which molecular resources and physiological data are available^14^,^15^. We exposed colonies of *S. pistillata* for more than 1 year to four pH treatments; pH 8, 7.8, 7.4 and 7.2 with corresponding Ω_*aragonite*_ values of 3.2, 2.3, 1.1 and 0.7, respectively. Although these acidification treatments extend well beyond ocean acidification scenarios predicted by the International Panel on Climate Change (IPCC) in the coming century, they are similar to treatments in previous investigations, for example^10^,^11^,^16^,^17^,^18^, that have proved useful in identifying clear trends in coral physiology under decreased pH/higher pCO_2_. We characterized the response of a broad suite of interrelated physiological and calcification parameters to these conditions. Our findings reveal that changes in coral calcification rate during CO_2_-driven seawater acidification can occur, not by physico-chemical dissolution of the skeleton, but rather by a change in skeleton morphology.

## Results

### Changes in skeletal growth parameters

**Figure 1:**
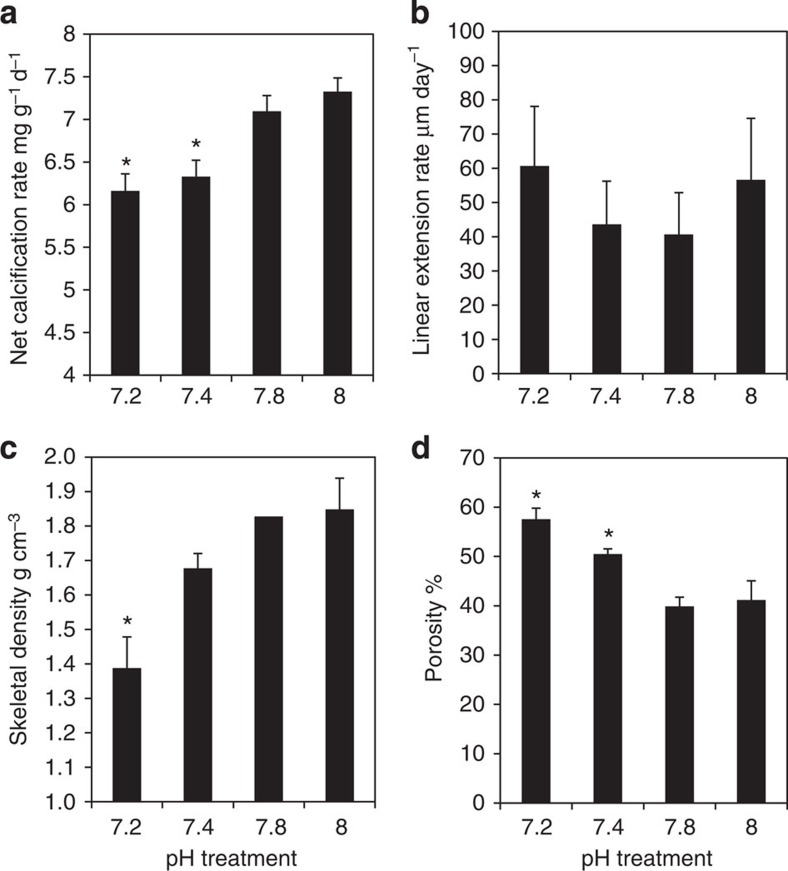
Skeletal growth parameters in the four pH treatments. (**a**) Net calcification rate (one way ANOVA, *n*=12, F_3_,_44_=4.11, *P*<0.05). (**b**) Linear extension (one way ANOVA, *n*=15, F_3_,_56_=0.62, *P*>0.05). (**c**) Bulk skeletal density (one way ANOVA, *n*=7, F_3_,_24_=16.44, *P*<0.001). (**d**) Skeletal porosity (one way ANOVA, *n*=3, F _3_,_8_=11.05, *P*<0.05). Data are means±s.e.m. Asterisk (*) indicates values that are significantly different for treatment with pH 8 (*P*<0.05).

Throughout the investigation, coral colonies remained visibly healthy (polyps remained extended and corals unbleached), and calcified in all experimental pH treatments, including treatment pH 7.2 (Ω_*aragonite*_<1)(carbonate chemistry of treatments given in Table 1). Respiration and photosynthetic rates of coral colonies remained unchanged across experimental treatments, with respiration rates higher than net photosynthetic rates in all treatments (Table 2). As anticipated, rates of calcification declined under seawater acidification, with significant decreases measured at pH 7.4 and 7.2 relative to calcification at pH 8 (Fig. 1a). By contrast, rates of linear extension of corals did not change significantly across pH treatments (Fig. 1b). Instead, skeletal bulk density decreased significantly at low pH (Fig. 1c) and quantitative x-ray micro-computed tomography CT (micro-CT) analysis revealed an increase in skeletal porosity at pH 7.4 and 7.2 relative to pH 8 (Fig. 1d). Together, these data show that declines in calcification rate are manifested in a change in skeletal density and porosity, and not in extension rates of the skeleton.

### Causes of increases in skeletal porosity

**Figure 2:**
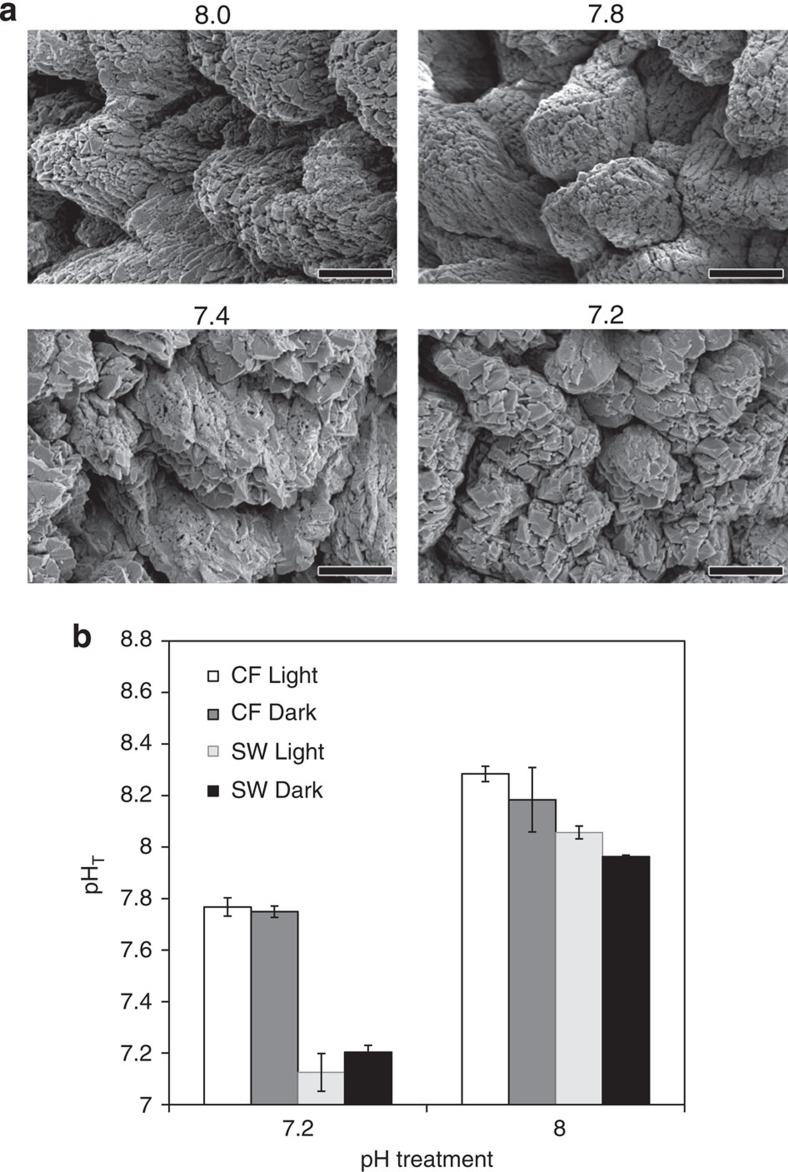
Indicators of physio-chemical conditions at the site of calcification in corals in the four pH treatments. (**a**) Aragonite crystal morphology imaged by scanning electron microscopy. Scale bar, 5 μm. pH treatment indicated above each image. (**b**) Calcifying fluid pH in corals under light and dark conditions at seawater pH 7.2 and pH 8.0. Data are means±s.e.m. CF=calcifying fluid; SW=seawater surrounding the colonies.

We endeavoured to determine the cause of increasing skeletal porosity, one possibility being that dissolution is responsible for such an effect. To investigate whether dissolution could occur in the coral's internal calcifying fluid where skeletal aragonite crystals form, we used confocal microscopy to measure calcifying fluid pH 11,19,20. Our calcifying fluid pH measurements were consistent with our earlier work on *S. pistillata*^11^, showing that, although seawater acidification depresses pH in the calcifying fluid to a certain extent, calcifying fluid pH remains high relative to the surrounding seawater (Fig. 2). Here we show for the first time, that calcifying fluid pH remains elevated in darkness as well as in light under conditions of seawater acidification (Fig. 2). These data are entirely consistent with our published estimates of Ω_*aragonite*_ of the calcifying fluid at similar pH values^11^, and indicate that the calcifying environment in *S. pistillata* is supersaturated with respect to aragonite in corals from pH 8 and 7.2 treatments in light and darkness. Dissolution due to decreases in pH at the site of calcification would therefore not be expected to be responsible for increases in skeletal porosity.

We also inspected aragonite crystal morphology in coral skeletons taken from the four pH treatments using scanning electron microscopy (SEM). We compared aragonite crystal morphology across treatments, on the skeleton surface (Fig. 2) and within pores inside the skeleton. In addition to daytime sampling, we also examined samples taken at the end of the night time period, to check whether dissolution was occurring in darkness (Supplementary Fig. 1). In all cases aragonite crystal morphology was very similar, comprising bundles of fibres that ranged between flat blades and rhomb-shaped structures, as observed previously for corals^21^,^22^. No signs of damage or dissolution were detected to the fibre-bundle arrangement nor to the smooth faces of the fibres. The crystal morphology we observed is unlike coral aragonite crystals that have undergone dissolution, which are irregular and corroded by comparison, as shown in previously published SEM images of coral skeleton dissolution^18^.

**Figure 3:**
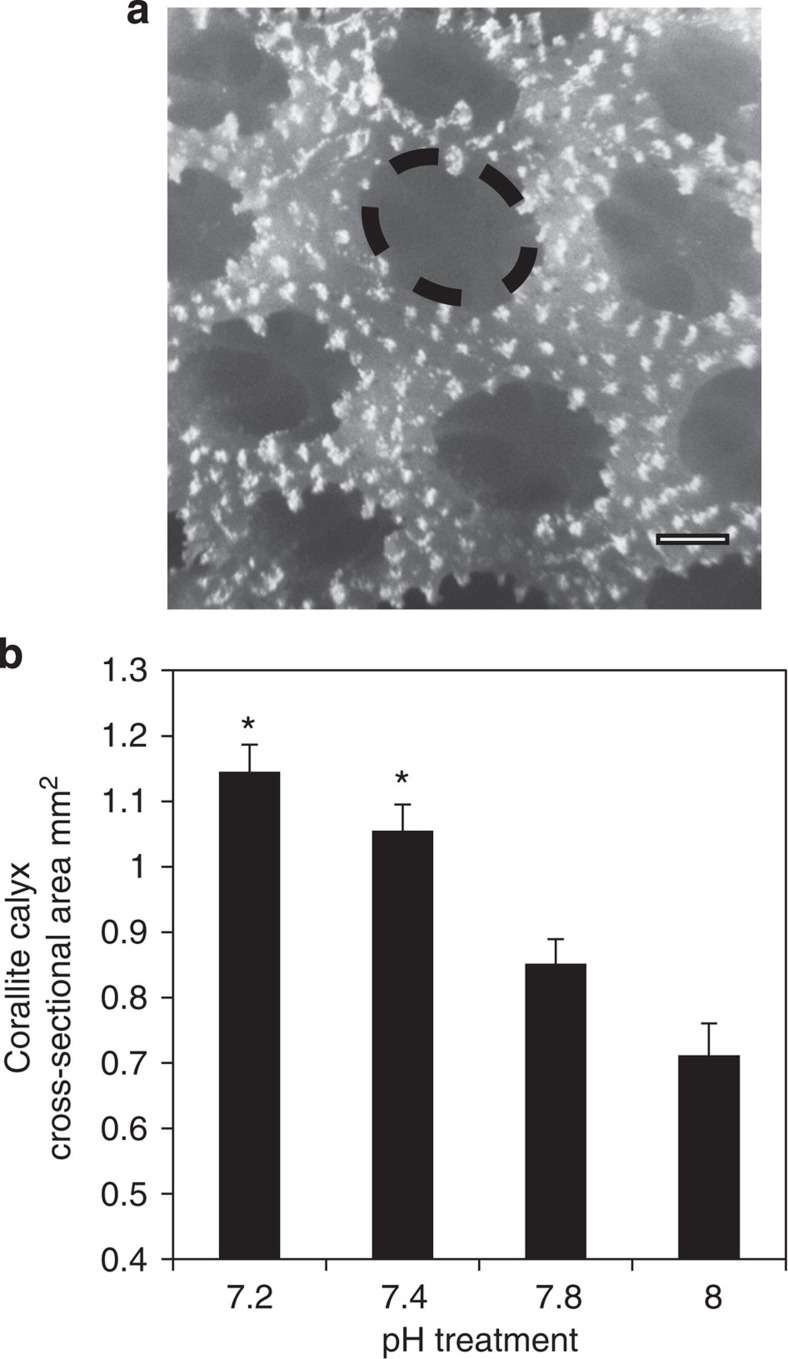
Corallite calyx size in the four pH treatments. (**a**) Image of corallite calyxes in the skeleton of *S. pistillata.* Dotted line shows the extent of cross-sectional area of a representative corallite calyx. Scale bar, 0.5 mm. (**b**) Corallite calyx size (cross-sectional area) (one way ANOVA, *n*=9, F_3_,_32_=21.60, *P*<0.001). Data are means±s.e.m. Asterisk (*) indicates values that are significantly different for treatment with pH 8 (*P*<0.05).

**Figure 4:**
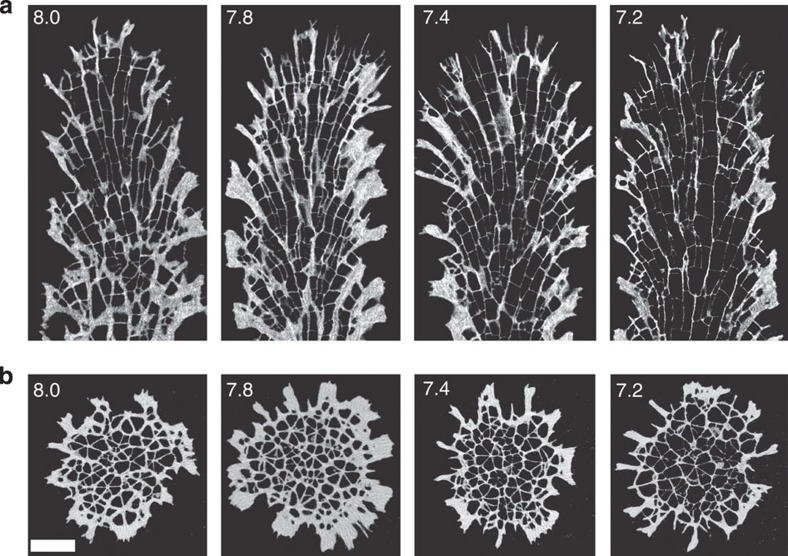
Coral skeleton morphology in the four pH treatments imaged by micro-CT. (**a**) Representative longitudinal sections; (**b**) transverse sections. pH treatment is indicated in the top left corner of each image. Scale bar, 1 mm.

An alternative explanation for the increase in skeletal porosity under acidification was found at higher levels of organization in the skeleton's architecture. We measured the size of corallite calyxes (the interior diameter of the cup-shaped openings in the skeleton that house the polyps^23^ (see Supplementary Fig. 2 for nomenclature of coral skeletal elements)). The size of corallite calyxes significantly increased with decreasing pH (Fig. 3a,b) and as larger corallite calyxes formed under acidification, there was a corresponding decrease in the number of corallites per unit surface area (Supplementary Fig. 3). Corallite calyx size from the four pH treatments was significantly correlated with both porosity and bulk density, indicating that the acidification-induced enlargement of corallite calyx size is a factor explaining the decrease in skeletal density and increase in porosity (Spearman' s rank-order tests: *r*_s_=0.783, *P*<0.01; *r*_s_=0.744, *P*<0.01). In addition, micro-CT images showed that enlargement of corallite calyxes at the surface of the skeleton corresponded to larger corallites extending throughout the interior of the skeleton, making it more porous (Fig. 4). Micro-CT images also revealed that corallites formed at lower pH are characterized by thinner septae, thecae and areas of coenosteum (Fig. 4), indicating that changes in the development of these features also contributed to changes in skeletal porosity. Taken together, these observations constitute an important finding: changes in calcification rate can be explained, not by physico-chemical dissolution of the skeleton, but rather by a change in skeleton phenotype.

### Organic matrix protein incorporation in the skeleton

**Figure 5:**
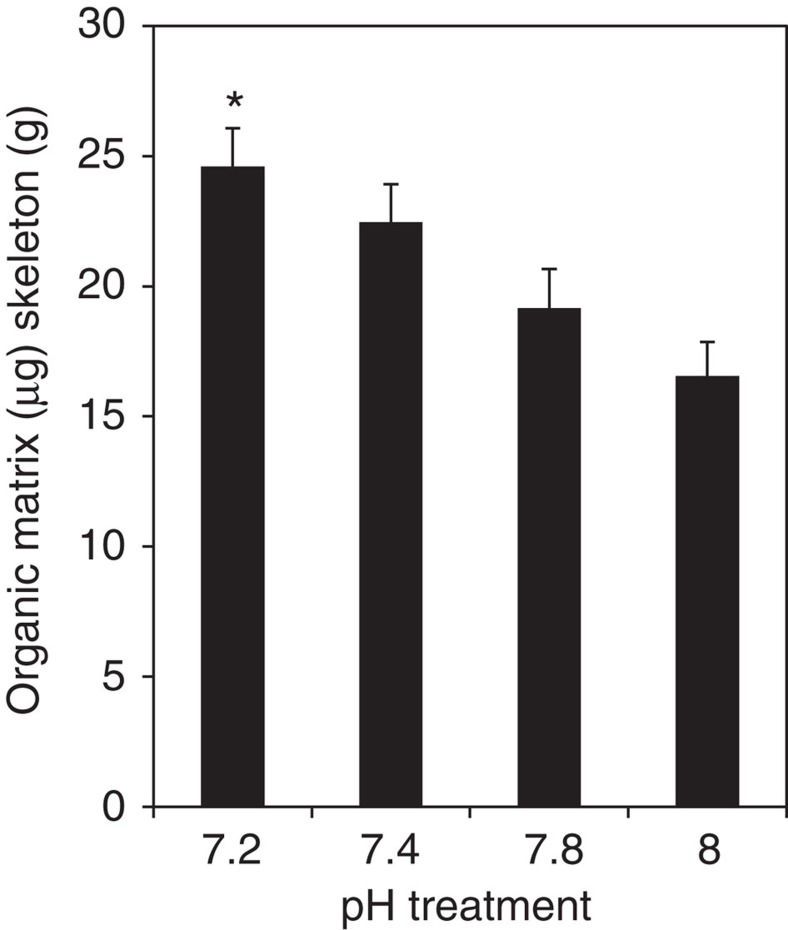
Organic matrix protein content of the coral skeletons in the four pH treatments. (one way ANOVA, *n*=6, F_3_,_20_=3.384, *P*<0.05). Data are means±s.e.m. Asterisk (*) indicates values that are significantly different for treatment with pH 8 (*P*<0.05).

Changes in skeleton morphology were accompanied by a significant increase in organic matrix (OM) proteins per gram of skeleton in corals from pH 7.2 versus pH 8 (Fig. 5). The increase in the ratio of OM proteins to gram of skeleton is not driven solely by the decrease in calcification, because, when OM per gram is converted to OM per cm^3^ using mean bulk density values (Fig. 1c), an increase in OM is still observed (30.60 μg cm^−3^ at pH 8 versus 34.16 μg cm^−3^ at pH 7.2). This finding is the first indication that ocean acidification causes an enrichment in OM incorporation in the coral skeleton and is also consistent with transcriptomic data from other coral species showing that the expression of certain OM protein genes are upregulated during acidification in coral juveniles^24^ and adults^17^.

## Discussion

The vulnerability of the coral calcification process to ocean acidification is well documented, but not well understood from a mechanistic point of view. As such, the current study aimed to investigate mechanisms underlying the calcification response of the model coral species *S. pistillata*, by analysing several interrelated calcification and physiological parameters. The principle finding of our study is that declines in coral calcification induced by seawater acidification were accompanied by a change in the morphology of the coral skeleton. At lower pH, corals were characterized by larger corallite calyxes and thinner associated skeletal structures including septae and thecae, resulting in more porous skeletons.

Recent field-based research has also noted increases in skeletal porosity associated with decreased pH and Ω_*aragonite*,_ but with the caveat that changes in skeletal growth may also have been affected by other environmental factors that co-vary in the field^25^. Here, in our laboratory experiments we were able to ensure that only pH and carbonate chemistry varied between our treatments, whereas other environmental factors were kept constant, enabling us to directly assign a role of seawater acidification in causing changes in skeletal formation. In addition, variation in rates of photosynthesis and respiration can be difficult to control in the field, but here under our experimental set-up, rates of these parameters were not different between the four treatments. This result is informative from a mechanistic perspective because it indicates that the calcification response we observed can be attributed to a direct effect of seawater acidification, rather than indirect effects via changes in photosynthetic or respiration rates.

In considering the mechanisms that contribute to changes in skeletal formation, dissolution could be considered as a potential factor, but our findings do not support this idea. Indeed SEM imaging of coral skeletons revealed similar aragonite crystal morphology in all the treatments, which is not consistent with aragonite crystals dissolving at lower pH treatments. This finding is in agreement with a previous study that demonstrated how living coral tissue protects the underlying skeleton from dissolution, even when corals are exposed for extended periods to seawater, which is under-saturated with respect to aragonite^18^. Given that our samples were entirely covered by tissue, dissolution would not be expected to be a factor contributing to changes in skeleton formation as long as pH and Ω_*aragonite*_ at the site of calcification is controlled.

Indeed, calcifying fluid pH measurements indicate that Ω_*aragonite*_ states were maintained at elevated levels in the calcifying environment in the lowest pH treatment, in both light and darkness. These data in the current study agree with a previous study that this species has a capacity to regulate calcifying fluid pH above seawater pH in the absence of photosynthesis^26^. This trait is also found in non-photosynthetic deep water coral species that have the capacity to maintain elevated calcifying fluid pH under decreased seawater pH^27^. The mechanisms by which corals achieve pH regulation of the calcifying fluid are not fully understood, but upregulation of calcifying fluid pH is believed to be an energy-requiring process that involves removal of protons from the calcifying fluid by Ca^2+^ATPases (reviewed in ref. 15). Available energy for pH upregulation is anticipated to be limited in darkness, as research with microsensors indicates that tissues near the skeleton are hypoxic in dark conditions in some corals^28^. Further research must better characterize pH regulation by calcifying (calicoblastic) cells and its energetics to reach a clearer understanding of this aspect of calcification physiology in a range of coral species. In any case, when determining the causes of changes in skeletal growth in the context of the current study, the key interpretation of the pH data presented here is that elevated calcifying pH values in the low pH treatment in dark or light conditions indicate that dissolution was not a factor leading to increasing porosity of coral skeletons.

Interestingly, we observed increased incorporation of OM proteins into the coral skeleton in corals grown under seawater acidification. This observation is intriguing considering that one of the primary roles of the OM in most biomineralizing systems is to reduce the free energy required for crystal nucleation, thereby facilitating calcification at lower saturation state values^29^. Recent research suggests that OM proteins do indeed perform this function in corals, as molecular characterization of OM proteins in *S. pistillata* and *in vitro* studies of their properties suggest that OM proteins catalyse precipitation of new aragonite crystals^12^. Given that corals exposed to reduced seawater pH experienced slight declines in the calcifying fluid pH and therefore Ω_*aragonite*_, it is possible that corals in the current study could have increased levels of OM proteins under acidification to promote calcification under less favourable calcifying fluid Ω_*aragonite*_. This is only a hypothesis, but future research into the role of the OM in coral biomineralization under conditions of seawater acidification could explore this possibility. Furthermore, in other marine calcifiers OM proteins have been found to direct orientation of aragonite crystals and determinate skeletal morphology^30^,^31^. These functions have not yet been demonstrated in corals, but the association of changes in skeletal morphology with the increase in OM proteins is intriguing and is also an area for future research.

**Figure 6:**
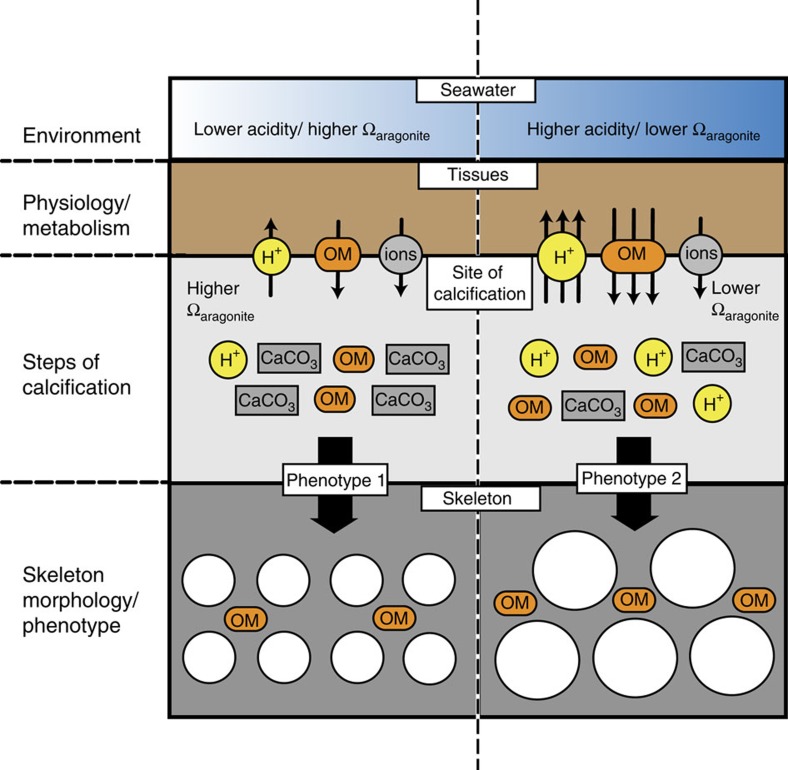
Schematic summary of the impact of ocean acidification on skeletal growth in *Stylophora pistillata*. Environmental change in the form of seawater acidification depresses pH and Ω_aragonite_ at the site of calcification. Coral physiology responds by increasing proton removal from the calcifying fluid to maintain elevated pH and Ω_aragonite_ that favours calcification. *S. pistillata* also increases production of organic matrix proteins (OM=organic matrix) per unit mass of CaCO_3_. In these conditions, corals continue to calcify, and dissolution of the skeleton does not occur, even when seawater Ω_aragonite_ <1. However, with lower saturation states in the calcifying fluid and increased energy expenditure for calcification, *S. pistillata* changes its skeleton phenotype to a morphology characterized by larger corallite calyxes. The resulting skeleton is more porous. ‘OM'=organic matrix. ‘Ions' represent both transcellular and paracellular transport of ions needed for the steps of calcification. White circles in skeleton represent corallites. ‘Steps of calcification' encompass steps of skeleton precipitation and assembly outlined in Tambutté *et al*.^15^.

We synthesized the results of our study to describe the phenotypic response of *S. pistillata* to seawater acidification (Fig. 6). Exposure of corals to seawater acidification results in increased rates of proton removal from the calcifying fluid to maintain pH elevated at the site of calcification^11^. Nevertheless, as the proton gradient with the surrounding seawater becomes less favourable, calcifying fluid pH (and therefore Ω_*aragonite*_) decreases slightly^11^. At the same time, *S. pistillata* increases production of OM proteins per unit mass of CaCO_3_. In these conditions, corals continue to calcify and dissolution of the skeleton does not occur, even when seawater Ω_*aragonite*_ <1. However, pH upregulation and OM production are energy-requiring processes; thus, calcification becomes more difficult and energetically more costly under seawater acidification. Rates of CaCO_3_ production decrease and *S. pistillata* changes the morphology of the skeleton, shifting to a skeletal phenotype characterized by larger corallite calyxes and thinner septae and thecae. The resulting skeleton is more porous and less dense, but rates of linear extension are maintained.

The potential ecological consequences of the trade-off of maintaining linear extension while producing a more porous skeleton are unknown, but it can be speculated that this may be a useful strategy for benthic organisms such as corals, where competition for space and light is an intense selective pressure^32^. However, as increasing skeletal porosity has been shown previously to result in more fragile skeletons^33^, this trade-off could present significant disadvantages. Fragile, porous skeletons are more susceptible to bioerosion^34^ and could be more easily damaged in high waveenergy environments or during events such as hurricanes^33^. Interestingly, our model species *S. pistillata* has been previously categorized as a species that is tolerant to ocean acidification over the same pH range tested here^10^,^11^,^16^. This is largely based on the fact this species effectively mitigates the effect of decreasing seawater pH and Ω_*aragonite*_ at the site of calcification by upregulating pH^10^,^11^. However, the present study demonstrates that a more comprehensive, multi-parameter assessment of skeleton formation under seawater acidification can reveal previously unseen vulnerabilities in the calcification process; in this case, a morphological shift to more porous and potentially more fragile skeletal architecture.

In any case, extrapolation of our laboratory-based study to predictions of how corals will behave in the field in a future high-CO_2_ world should be made with caution. While informative from a mechanistic point of view, our study was conducted inside narrowly controlled environmental parameters, which do not simulate the dynamic and changing environment on the reef. For example, corals living in shallow water can experience greater midday irradiances than levels provided in the current experiments. It is possible that the combination of higher irradiances with seawater acidification may have resulted in additional effects on coral physiology, notably bleaching (loss of algal symbionts), as observed in previous work^35^. Furthermore our mechanistic study has relied on a single, model species that is tractable for laboratory studies, but the calcification and associated physiology of other coral species may respond differently to seawater acidification. Notable examples include previous accounts of where ocean acidification has caused some coral species to change from colonial forms to non-calcifying single polyp forms^36^.

In conclusion, our study on *S. pistillata* reveals how CO_2_-driven seawater acidification can cause a shift in coral skeleton morphology that occurs in concert with a decrease in coral calcification rates. The novelty of these findings lies in the demonstration that seawater acidification not only decreases the rate at which coral skeletons grow, but it can also affect assembly of the overall skeletal architecture. The porous-phenotype that we describe here may potentially lead to more fragile coral skeletons under conditions of acidification making corals more vulnerable to damage and bioerosion. More generally, our findings underline the importance of using a multi-parameter analysis of skeletal growth when investigating the vulnerability of coral species to ocean acidification.

## Methods

### Biological material and treatments

Colonies of the tropical coral *Stylophora pistillata* were exposed to long-term seawater acidification as described previously^11^. Corals were kept in aquaria supplied with Mediterranean seawater (exchange rate 70%/h) at a salinity of 38, temperature 25 °C and irradiance of 170 μmol photons m^−2^ s^−1^ on a 12-h/12-h photoperiod provided by HQI-10000K metal halide lamps (BLV Nepturion). Carbonate chemistry was manipulated by bubbling with CO_2_ to reduce pH to the target values of pH 7.2, pH 7.4 and pH 7.8 (Table 1). A fourth treatment (pH 8) was not bubbled with CO_2_ (Table 1). pH and temperature probes (Ponsel-Mesure, France) were installed in the four treatment aquaria and connected to a custom-made monitoring system (Enoleo, Monaco), which controlled CO_2_ bubbling. On a daily basis, pH checks were carried with a Digital ODEON pH meter (Ponsel-Mesure, France). All probes were calibrated to pH total scale. In addition, biweekly pH measurements were made using the indicator dye m-cresol purple (Acros 199250050) adapted from Dickson *et al*.^37^; the absorbance was measured using a spectrophotometer (UVmc2; Safas, Monaco).

Measurements of total alkalinity (TA) were made weekly according to protocols described in Dickson *et al*.^37^. TA was measured via titration with 0.03 N HCl containing 40.7 g NaCl per litre using a Metrohm Titrando 888 Dosimat controlled by Tiamo software to perform automated titrations of 4-ml samples, and alkalinity was calculated using a regression routine based on Department of Energy guidelines^38^. For each sample run, certified seawater reference material supplied by the laboratory of A. G. Dickson (Scripps Institution of Oceanography, La Jolla, CA) was used to verify acid normality. Parameters of carbonate seawater chemistry were calculated from total scale pH, TA, temperature and salinity using the free-access CO2SYS package (10) using constants from Mehrbach *et al*.^39^ as refit by Dickson and Millero^40^. Parameters of carbonate seawater chemistry in each treatment are given in Table 1.

In each of the four aquariums, submersible pumps ensured high water circulation. Corals were fed twice a week with *Artemia salina* nauplii. To avoid ‘tank' effects, aquariums were rigorously cleaned every week to prevent the growth of epiphytic algae and fouling communities or the accumulation of detritus. Biomass in the aquariums was kept relatively constant by sampling and by addition of new corals (coral colonies were identified by labelling colonies with a radio frequency identification microchip). This maintenance, the high seawater renewal, and the regular monitoring of seawater chemistry (described below), temperature and irradiance ensured that similar conditions prevailed in each aquarium, except for the carbonate chemistry.

### Experimental design and statistical analysis

Analysis of physiological and calcification parameters was conducted on coral colonies that had already experienced 1 year of exposure to the pH treatments. Analysis of each parameter was carried out in sequential experimental trials. Each trial included replicates from each of the four treatments. Sample sizes for each parameter maximized replication while keeping experiments feasible. The number of replicates and trials for each parameter are given in the following text.

For each experimental parameter, results of the experimental trials were compared using two-way analysis of variance (ANOVA) using trial and pH treatment as factors. If no significant difference was found between the experimental trials or if the trend of the response to acidification was identical in trials, then the results of the trials were pooled and analysed together using one-way ANOVA, with Student-Newman-Keuls *post hoc* analysis to identify significant differences between treatments. Correlation analysis was carried out by using Spearman rank-order tests. Where necessary, the data were logarithmically or square-root transformed to adhere to the assumptions necessary for parametric analysis (normal distributions with homogenous variances). Percentage data were arcin-square root transformed for normalization. Data were analysed with SPSS Statistics 21 software (IBM, France).

### Calcification rate

Net calcification rate was measured by buoyant weight analysis^41^. Working with microcolonies grown suspended on nylon threads, corals were suspended below a microbalance (XP205 delta range, Mettler Toledo, France) in seawater maintained at 25 °C. Corals were buoyant weighed in this manner weekly for 20 weeks. Net calcification rate was determined by normalizing the change in buoyant weight of each sample by the initial buoyant weight of the sample and then dividing by the number of days between sampling times. Data were thus expressed as mg g^−1^ d^−1^.

The analysis was carried out in two experimental trials using six microcolonies per treatment each trial.

### Linear extension

Linear extension was assessed by measuring the increase in height of branches attached to glass slides. Branch apexes ∼1 cm high were attached to glass slides and allowed to recover. Calipers were then used to measure the height of the colony, and measurements repeated 2–3 months later. The analysis was carried out in five experimental trials with three replicates per treatment each trial.

### Skeletal bulk density

Bulk density was obtained by methods adapted from Bucher *et al*.^42^. Branches of similar size were sampled from seven colonies in each pH treatment and placed in 10% NaClO to remove tissues. Branches were then rinsed and oven-dried to constant weight and weighed to obtain clean dry weight (*DW*_clean_). Samples were then coated with paraffin wax and the dry weight (*DW*_wax_) measured again. The buoyant weight (*BW*_wax_) was obtained in dH_2_O at 20 °C. Total enclosed volume was calculated by the following formula:


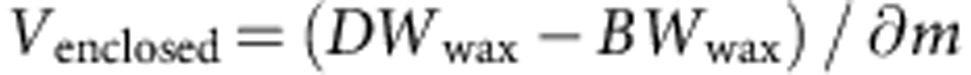


Where ∂m is the density of the medium (that is 1g cm^3^). The bulk density was determined by the following equation:


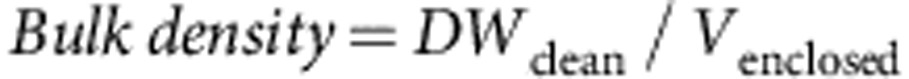


Bulk density was analysed in one experimental trial, with seven replicates per treatment.

### Skeletal porosity

X-Ray micro-computed tomography (Micro-CT) is a non-invasive high-resolution imaging method for visualizing the external and internal structure of objects that has recently been validated to examine porosity in coral skeletons^43^,^44^. Micro-CT analysis was carried out at the Polyclinique St Jean, Cagnes sur Mer, France, with an Skyscan 1173 compact micro-CT (SkyScan, Antwerp, Belgium). A microfocus X-ray tube with a focal spot of 10 μm was used as a source (80 kV, 100 μA). The sample was rotated 360° between the X-Ray source and the camera. Rotation steps of 1.5° were used. At each angle an X-Ray exposure was recorded on the distortion-free flat-panel sensor (resolution 2,240 × 2,240 pixels). The resulting slice is made of voxels, the three-dimensional equivalent of pixels. Each voxel is assigned a grey value derived from a linear attenuation coefficient that relates to the density of materials being scanned. All specimens were scanned at the same voxel size. The radial projections were reconstructed into a three-dimensional matrix of isotropic voxels ranging from 5 to 10 μm, depending on the exact height of the coral tip.

X-Ray images were transformed by NRecon software (Skyscan) to reconstruct 2-D images for quantitative analysis. From these 2-D images evaluation of the morphometric parameters was performed using CTann®CT analysis software (SkyScan). A manual greyscale threshold was implemented manually on the first set of images and then applied to all specimens.

For each sample, a digital region of interest was created to extend through 100 μm of skeleton at 7 mm distance from the apex corresponding to about 15 slices. Then the percentage negative space versus skeleton was determined, providing the measure of porosity.

For each treatment condition, three branches of similar size were taken from the apical part of a colony. Porosity was analysed in one experimental trial with three replicates per treatment.

### Calyx size (interior diameter of corallites)

Branches of similar size were sampled from colonies in each pH treatment, and placed in a 10% NaClO solution for 30 min to remove tissues. Skeletons were then rinsed several times in ultrapure H_2_O and dried at room temperature. Samples were observed under a macroscope at × 15 magnification (Z16APO, Leica Microsystems) using a digital camera (Tri-CCD JAI AT200 GE 2MP) to determine calyx size and density. The interior diameter of corallites (which we call calyx according to the terminology of (Johnson 1981)) was determined by measuring the cross-sectional area of all calyxes per branch and obtaining the mean. Corallite calyx density was calculated by normalizing the total number of calyxes on the branch to its surface area. Cross-sectional area measurements and counting of calyxes were achieved using SAISAMsoftware (Microvision Instruments, France). Calyx size and density were assessed in three experimental trials with three replicates per trial.

### SEM of skeleton microstructure

Skeleton microstructure (for example, crystal morphology) was examined in three colonies from each treatment at the end of daytime and night time periods by SEM as described previously in Tambutté *et al*.^45^. In brief, the soft tissues were removed with NaClO 10%, rinsed with distilled water then oven-dried. All samples were coated with gold and observed at 5 kV in a JEOL JSM-6010LV. Analysis of skeleton microstructure was carried out in one experimental trial.

### Confocal microscope measurements of calcifying fluid pH

Analysis was carried out as described previously^11^ on samples grown laterally on glass coverslips fitted in semi-closed perfusion chambers (PeCon, Germany) that were mounted on the confocal microscope and supplied with seawater drawn from the desired acidification treatment. The seawater pH and carbonate chemistry of the perfused seawater was checked by measuring TA and pH in the inflowing and outflowing seawater to check that carbonate chemistry did not drift away from the target values in treatment aquariums during the period of measurement. We used higher flow rates than in previous work (for example, ref. 11) to achieve stable pH and carbonate chemistry in both light and dark conditions (renewal rate of 50% per min of a 2.5-ml vol). Sample sizes were restricted to 1 cm^2^ in surface area, irradiance provided at 170 μmol photons m^−2^ s^−1^ (Philips 21V 150-W halogen bulb) in light treatments, and temperature maintained at 25 °C. Measurement of oxygen in seawater in the perfusion chamber with a needle-type microsensor (PreSens, Germany) in light and darkness indicated oxygen levels also remained stable between values of 265–280 μmol l^−1^ under these conditions.

### Dye loading and pH measurements

Measurements of pH of the calcifying fluid (=subcalicoblastic medium) in the light and dark were made by inverted confocal microscopy (Leica SP5, Germany) and the ratiometric dye SNARF-1 (Invitrogen) according to methods we published previously (refs 11, 26).

Before pH measurements, samples were first perfused with seawater from the desired pH treatment for 10 min in either light or darkness. Samples were then loaded with pH-sensitive dye by perfusion of seawater containing 45 μM cell-impermeable SNARF-1 for an additional 5 min under the same conditions. Perfusion of samples with seawater at the desired pH treatment containing SNARF-1 continued for an additional 10 min in light or darkness, during which five measurements of calcifying fluid pH were made 2 min apart to ensure a stable pH value was obtained.

Calcifying fluid pH measurements were carried out at × 40 magnification by excitation of SNARF-1 at 543 nm at 30% laser intensity and fluorescence captured at emission wavelengths of 585±10 nm and 640±10 nm. For each measurement, several optical sections were captured in a *Z*-stack, with an acquisition time of ∼10 s, during which the part of the colony under analysis is exposed to the laser. The ratio of fluorescence at the emission wavelengths was calibrated to pH by methods previously published^26^, except that the standard curves were produced using seawater adjusted to the range pH 7–9 on total scale rather than National Bureau of Standards (NBS) scale.

Calcifying fluid pH was measured in light and dark conditions in three samples from pH treatments pH 7.2 and pH 8.

### OM protein content

Branches were sampled and incubated in 10% sodium hypochlorite (NaClO) to eliminate soft tissues and separate skeletons. Skeletons were thoroughly rinsed with ultrapure water and cryo-ground (Spex SamplePrep 6770 apparatus) into powder of homogeneous granulometry (about 30 μm diameter). The powder was incubated with 10% NaClO at 4 °C for 24 h to remove potential contaminants such as endoliths. The resulting solution was centrifuged (3,500 g, 5 min, 4 °C). The pellets of skeleton powders were rinsed several times with ultrapure water and freeze dried. To separate the organic fraction from the mineral fraction, 15 g of powder of skeletons were demineralized in 0.25 M EDTA (pH 7.8, 23 h, 4 °C), the solution was prefiltered on 0.2 μm of polyethersulfone filters and then filtered in tandem through two Sep-Pak Plus C18 cartridges (Waters, 5 kDa) according to the protocol of Rahman *et al*., 2013. The eluted macromolecules were frozen at −80 °C and subsequently lyophilized. Protein content was determined using the bicinchoninic acid assay kit (BC Protein Assay, Interchim). The standard curve was established with bovine serum albumin and the absorbance was measured with a microplate reader (EpochTM, Bioteck, US) at 562 nm.

The analysis was carried out in two experimental trials with three replicates per treatment for each trial.

### Photosynthesis and respiration rates and biomass parameters

Five microcolonies of similar size were used for measuring photosynthetic rates and respiration, after which they were frozen at –80 °C. Protein content, chlorophyll (Chl) content and zooxanthella density were determined as described below.

Each microcolony was placed on a nylon net in a closed beaker agitated using a magnetic stirrer. Incubations were performed in the same conditions of temperature (25 °C) and seawater chemistry as in experimental tanks but either under light conditions (170 μmol photons m^−2^ s^−1^) for photosynthesis, or dark conditions for respiration (seawater pH and alkalinity were checked at the end of each experiment). An oxygen optode sensor system (oxy-4 mini, PreSens, Regensburg, Germany) was used to quantify oxygen flux. Data were recorded with OXY4v2_11FB software (PreSens). Before each measurement, the oxygen sensor was calibrated against air-saturated seawater (100% oxygen) and a saturated solution of sodium sulfite (zero oxygen). Rates of photosynthesis and respiration were estimated by regressing oxygen data against time and normalized to surface area, protein content or symbiont cell. Photosynthesis and respiration rates were measured in three experimental trials, with five replicates per treatment each trial.

Frozen samples were placed in filtered seawater and tissues removed with a jet of pressurized nitrogen. The skeleton was collected, rinsed in distilled water, oven-dried for 24 h at 100 °C and used for measuring surface area. The tissues were homogenized with a Potter grinder and divided into three aliquots, one for measuring protein content, one for measuring chlorophyll content and one for measuring symbiont density.

For protein content, homogenized tissues in filtered seawater were centrifuged and the pellet suspended in 1 M NaOH at 90 °C for 10 min. Protein content was determined using the bicinchoninic acid assay kit (BC Protein Assay, Interchim). The standard curve was established with bovine serum albumin and the absorbance was measured with a microplate reader (EpochTM, Bioteck, US) at 562 nm. Data were normalized to skeleton surface area. For chlorophyll pigment content, homogenized tissues in filtered seawater were centrifuged and the pellet suspended in 100% acetone at 4 °C for 24 h. The extraction was repeated and the extracts were pooled and centrifuged at 10,000 *g* for 15 min. Absorbance of extracts were measured at 630 and 663 nm in a spectrophotometer (UVMC2, Safas, Monaco) against an acetone blank. Concentrations of chlorophyll a and c_2_ were calculated using the equations of Jeffrey and Humphrey^46^.

For symbiont density, counting was performed using the HistolabH 5.2.3 image analysis software (Microvision Intruments, rance) (ref. 47).

Surface area of colonies was measured utilizing one of the common methods currently used, the paraffin wax method, Stimson and Kinzie 1991 (ref. 48). In brief, coral skeletons were coated in paraffin wax (Paraplastwax—Sigma, France) at 65 °C. Surface area of the specimens was obtained by referring the weight of the paraffin wax coated on the specimen to the standard curve of paraffin wax versus surface area. The standard curve was generated by regressing weight of the paraffin wax to known surface area density blocks.

## Additional information

**How to cite this article**: Tambutté, E. *et al*. Morphological plasticity of the coral skeleton under CO_2_-driven seawater acidification. *Nat. Commun.* 6:7368 doi: 10.1038/ncomms8368 (2015).

## Supplementary Material

Supplementary Information Supplementary Figures 1-3.

## Figures and Tables

**Table 1:** Carbonate chemistry parameters in the four experimental pH treatments.

**Treatment name**	**pH**_**T**_	**Total alkalinity (μmol kg**^**−1**^**-SW)**	**pCO**_**2**_ **(μatm)**	**HCO**_**3**_^−^ **(μmol kg**^**−1**^**-SW)**	**CO**_**3**_^**2**−^ **(μmol kg**^**−1**^**-SW)**	**Total carbon (μmol kg**^**−1**^**-SW)**	Ω**ar**
pH 7.2	7.2±0.01	2530.96±7.15	3792.57±52.54	2423.67±5.22	44.23±0.81	2573.68±4.57	0.69±0.01
pH 7.4	7.40±0.01	2490.00±6.06	2256.88±32.98	2323.49±3.20	68.38±1.21	2454.76±3.48	1.06±0.02
pH 7.8	7.79±0.01	2485.12±5.25	856.17±13.63	2119.11±1.40	149.94±2.37	2292.90±1.58	2.33±0.04
pH 8.0	7.95±0.00	2461.73±6.31	538.41±4.85	1961.44±1.48	204.27±2.06	2180.71±3.42	3.17±0.03

Data are means±s.d. Parameters of carbonate seawater chemistry were calculated from total scale pH, TA, temperature and salinity using the free-access CO2SYS package (ref. 10) using constants from Mehrbach *et al*.^39^ as refit by Dickson and Millero^40^.

**Table 2:** Physiological parameters measured in the four pH treatments.

	**Units**	**7.20**	**7.40**	**7.80**	**8.0**	**F**	***P*****-value**
Normalized by surface area							
Photosynthesis	μmol O_2_ h^−1^ cm^−2^	1.14±0.18	0.82±0.15	1.00±0.18	1.01±0.23	F_3_,_36_=0.988	0.409
Respiration	μmol O_2_ h^−1^ cm^−2^	1.39±0.24	1.31±0.23	1.33±0.20	1.25±0.18	F_3_,_36_=0.072	0.974
Protein	mg cm^−2^	1.18±0.12	0.91±0.11	0.95±0.11	0.97±0.10	F_3_,_36_=2,493	0.76
Symbiont density	cells cm^−2^	2.11 × 10^6^±1.68 × 10^5^	1.78 × 10^6^±1.68 × 10^5^	1.50 × 10^6^±1.78 × 10^5^	2.14 × 10^6^±2.03 × 10^5^	F_3_,_16_=2.110	0.139
Chla	μg cm^−2^	9.77±0.58	11.20±0.59	9.35±0.58	7.80±1.05	F_3_,_16_=1.710	0.205
Chlc_*2*_	μg cm^−2^	2.01±0.16	2.27±0.16	1.63±0.16	1.74±0.12	F_3_,_16_=2.458	0.100
							
Normalized by protein							
Photosynthesis	μmol O_2_ h^−1^ mg^−1^	0.95±0.11	0.82±0.18	0.95±0.18	0.92±0.14	F_3_,_48_= 0.444	0.723
Respiration	μmol O_2_ h^−1^ mg^−1^	1.03±0.21	1.40±0.36	1.45±0.21	1.24±0.12	F_3_,_48_= 0.554	0.648
Symbiont density	Cell mg^−1^	2.12 × 10^6^±1.53 × 10^5^	1.82 × 10^6^±1.72 × 10^5^	1.71 × 10^6^±1.54 × 10^5^	2.56 × 10^6^±1.58 × 10^5^	F_3_,_16_=0.699	0.566
Chl*a*	μg mg^−1^	9.82±0.51	11.44±0.59	10.39±0.72	9.41±1.16	F_3_,_16_=0.851	0.486
Chl*c*_*2*_	μg mg^−1^	2.01±0.14	2.02±0.16	2.20±0.18	2.34±0.24	F_3_,_16_=1.933	0.165
							
Normalized per symbiont cell							
Photosynthesis	μmol O_2_ h^−1^ cell^−1^	4.15 × 10^−7^±2.26 × 10^−8^	3.97 × 10^−7^±2.50 × 10^−7^	3.66 × 10^−7^±2.23 × 10^−8^	3.89 × 10^−7^±3.13 × 10^−8^	F_3_,_16_=2.493	0.967
Chl*a*	pg cell^−1^	4.69*±0.30	4.95*±0.28	5.03*±0.10	5.42±0.14	F_3_,_16_ =6,173	0.005
Chl*c*_*2*_	pg cell^−1^	0.96±0.06	0.99±0.05	1.04±0.05	1.09±0.05	F_3_, _16_=3,152	0.052

Data are mean±s.e. Data were analysed by 1 way ANOVA.

^*^Denotes mean values significantly different than the mean value from treatment with pH 8 (*P*<0.05).
