# Supplementary material for: Morphological plasticity of the coral skeleton under CO2-driven seawater acidification
Source: Nat Commun. 2015 Jun 12;6:7368. doi: 10.1038/ncomms8368 (PMC4490415; doi:10.1038/ncomms8368)
Supplement: Supplementary Information — Supplementary Figures 1-3. [file ncomms8368-s1.pdf]

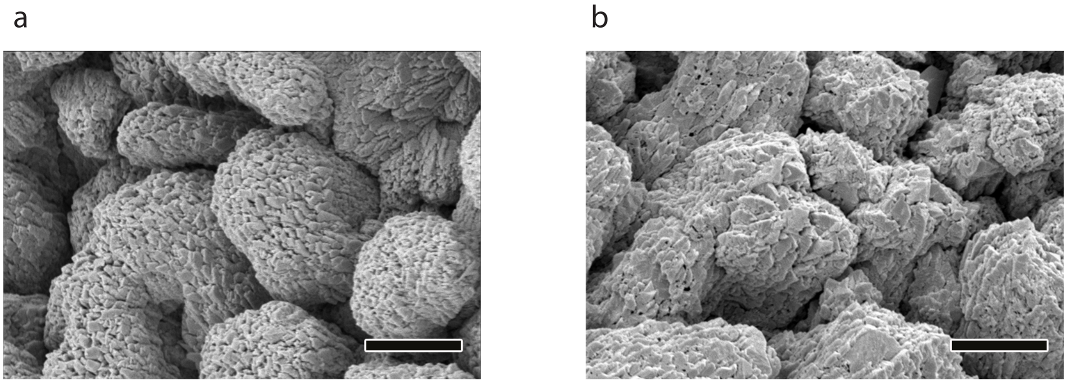

Supplementary Figure. 1. Scanning electron micrographs of aragonite crystals in the skeleton of *Stylophora pistillata.* a) and b) Aragonite crystals in samples after 12 h in darkness (end of night-time period) in treatments pH 8.0 and pH 7.2 respectively. Scale bar = 5µm.

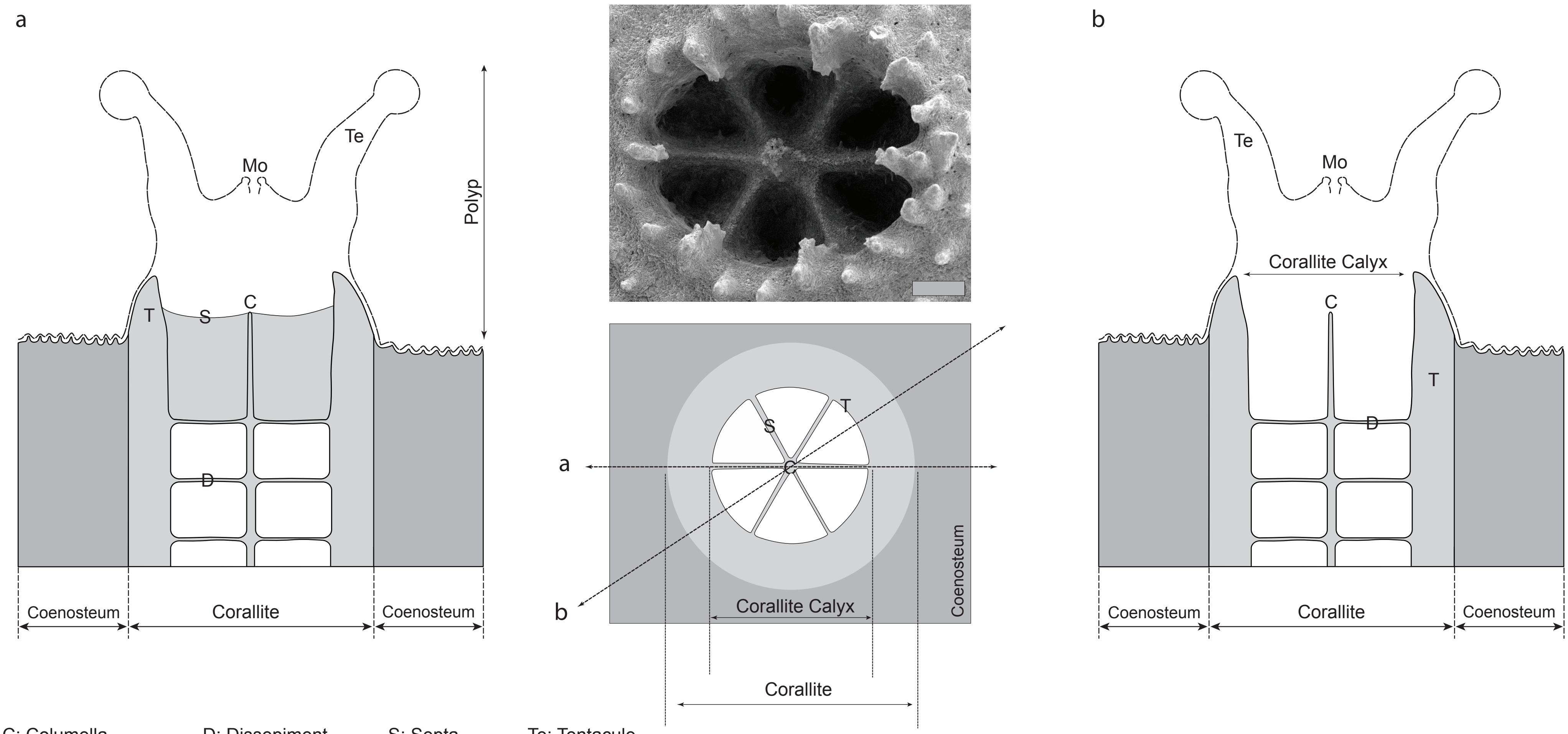

C: Columella
Co: Coenosteum
D: Dissepiment
Mo: Mouth
S: Septa
T: Theca
Te: Tentacule

Supplementary Figure 2. Nomenclature of structures in the coral skeleton. a and b are cross sections of the corallite calyx at different positions. Scanning electron micrograph shows a corallite of *Stylophora pistillata* (scale bar = 200 microns).

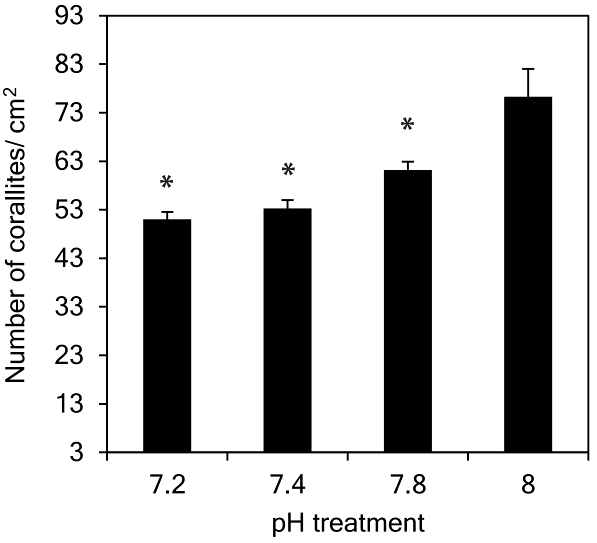

Supplementary Figure 3. Number of corallites normalized to skeleton surface area (one way ANOVA, n= 9, $F_{3,32}$ = 14.50.6, P < 0.001). Data are means and SEM. * denotes values that are significantly different to pH 8 (P < 0.05).
